# Determination of Critical Micelle Concentration of Ionic and Non‐Ionic Surfactants by Streaming Potential Measurements

**DOI:** 10.1002/elps.8145

**Published:** 2025-04-23

**Authors:** Yuri Chenyakin, David Da Yong Chen

**Affiliations:** ^1^ Department of Chemistry University of British Columbia Vancouver British Columbia Canada

**Keywords:** capillary electrophoresis apparatus, critical micelle concentration, streaming potential, surface charge density, surfactant, zeta potential

## Abstract

A capillary electrophoresis system capable of measuring streaming potentials was used for the determination of critical micelle concentration (CMC) of anionic, cationic, zwitterionic and non‐ionic surfactants. The CMC values of anionic surfactant sodium dodecyl sulphate (SDS), cationic surfactant cetyltrimethylammonium bromide (CTAB), zwitterionic surfactant 3‐((3‐cholamidopropyl) dimethylammonio)‐1‐propanesulfonate (CHAPS) and non‐ionic surfactant polyethylene glycol *p*‐(1,1,3,3‐tetramethylbutyl)‐phenyl ether (Triton X‐100) in water or salt solutions were determined by determining the abrupt change in the trend of streaming potential change with the surfactant concentration. The CMC values were 8.23, 0.93, 5.80 and 0.16 mM, respectively. This method was also used to demonstrate how the CMCs of SDS and CTAB change differently with temperature. The CMC of SDS decreased from 10°C to 25°C and then increased from 25°C to 40°C, whereas CTAB only increased linearly within 10°C–40°C. The capillary wall zeta potentials in surfactant solutions can be calculated from the measured streaming potential, conductivity and solution viscosity. Surface charge densities were calculated using the zeta potentials obtained. The surface charge densities of SDS were calculated to be 5.6–0.8 C/m^2^ when SDS solutions with concentrations of 2–20 mM zeta potentials were used. The calculated zeta potentials and surface charge densities reached a plateau at about 8 mM, which coincided with the CMC of SDS determined in the present study and the literature values. The CMC values obtained from streaming potential measurement are comparable to values obtained with other CMC determination techniques such as surface tension and conductometric measurements.

## 1 Introduction

Surface‐active agents, or more commonly known as surfactants, can self‐assemble in aqueous solutions into organized clusters known as micelles [1, 2, 3, 4, 5, 6, 7, 8, 9]. The specific concentration of the surfactant at which surfactant molecules become more favourable to aggregate into micelles in the bulk solution is called the critical micelle concentration (CMC). The CMC of a surfactant in an aqueous solution can be determined by the observation of a sudden change of certain physical properties of the surfactant solution when micelles begin to form. Methods such as surface tension or conductivity measurement, fluorometry and spectrophotometry can be used for the determination of CMC [10, 11, 12, 13, 14, 15, 16, 17, 18, 19]. Obtaining a minimum in interfacial tension or a maximum in detergency using particular surfactant often requires the knowledge of the CMC value [20, 21]. In this work, we introduce a new method to measure the CMC using a capillary electrophoresis (CE) apparatus capable of measuring streaming potentials. Streaming potential is the potential difference across a capillary tube when an electrolyte solution is forced through from the inlet to the outlet with a pressure [22, 23, 24]. Streaming potential measurements in laboratory settings are often performed using in‐house custom‐built equipment with a few exceptions that utilize commercial equipment [25, 26, 27, 28]. Using our CE apparatus, we showed that measuring streaming potentials of ionic (anionic, cationic and zwitterionic) and non‐ionic surfactant solutions can be used to determine the CMC value of surfactants in aqueous solutions at different temperatures. Also, we showed that the measured streaming potentials of surfactant solutions can be used to obtain important physicochemical parameters such as surface zeta potentials and surface charge densities.

## 2 Materials and Methods

### 2.1 Instrument

A Capel‐205 CE system (Lumex Instruments Canada, Mission, BC) was used for this experiment. The capillary used had a 50 µm inner diameter, 360 µm outer diameter and was 50 cm in length (43 cm to the 190 nm UV absorption detector). The uncoated fused silica capillary was purchased from Polymicro Technologies (Phoenix, Arizona) and cut to length in house. To precondition a new capillary, the fused silica capillary was cleaned by pushing 0.1 M NaOH aqueous solution through with a 1000 mbar pressure for 3 min, followed by a 1‐min rinse with deionized distilled water. The capillary was then rinsed with appropriate surfactant solution for 3 min using 1000 mbar and equilibrated with the same surfactant solution before being used for streaming potential measurements.

### 2.2 Chemicals

All chemicals were analytical grade and were purchased from Sigma‐Aldrich (Oakville, ON, Canada). Sodium dodecyl sulphate (SDS) solutions of 2, 4, 6, 8, 10, 12, 14, 16, 18 and 20 mM; cetyltrimethylammonium bromide (CTAB) solutions of 0.2, 0.4, 0.6, 0.8, 1, 2, 4, 6, 8 and 10 mM; 3‐[(3‐cholamidopropyl)dimethylammonio]‐1‐propanesulfonate (CHAPS) solutions of 2, 3, 4, 6, 8, 10, 12 and 14 mM and Triton X‐100 (C_14_H_22_O(C_2_H_4_O)*n*, high purity) solutions of 0.05, 0.1, 0.15, 0.2, 0.3, 0.4, 0.5, 0.6 and 0.7 mM were used in the experiment. Triton X‐100 is a non‐ionic surfactant that has a hydrophilic polyethylene oxide chain, on average 9.5 ethylene oxide units, and an aromatic hydrocarbon group.

### 2.3 Surfactant Sample

**FIGURE 1:**
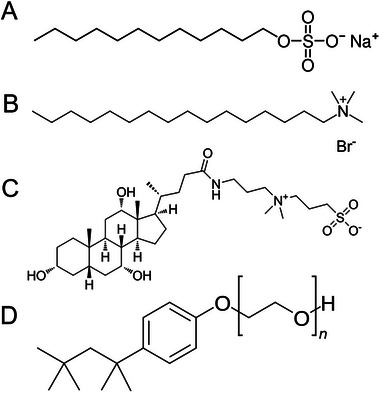
Chemical structures of (A) SDS, (B) CTAB, (C) CHAPS and (D) Triton X‐100.

The surfactant solutions were prepared by dissolving solid compounds in deionized distilled water and diluted to desired concentrations at room temperature. The chemical structures are depicted in Figure 1.

### 2.4 Streaming Potential Measurement

**FIGURE 2:**
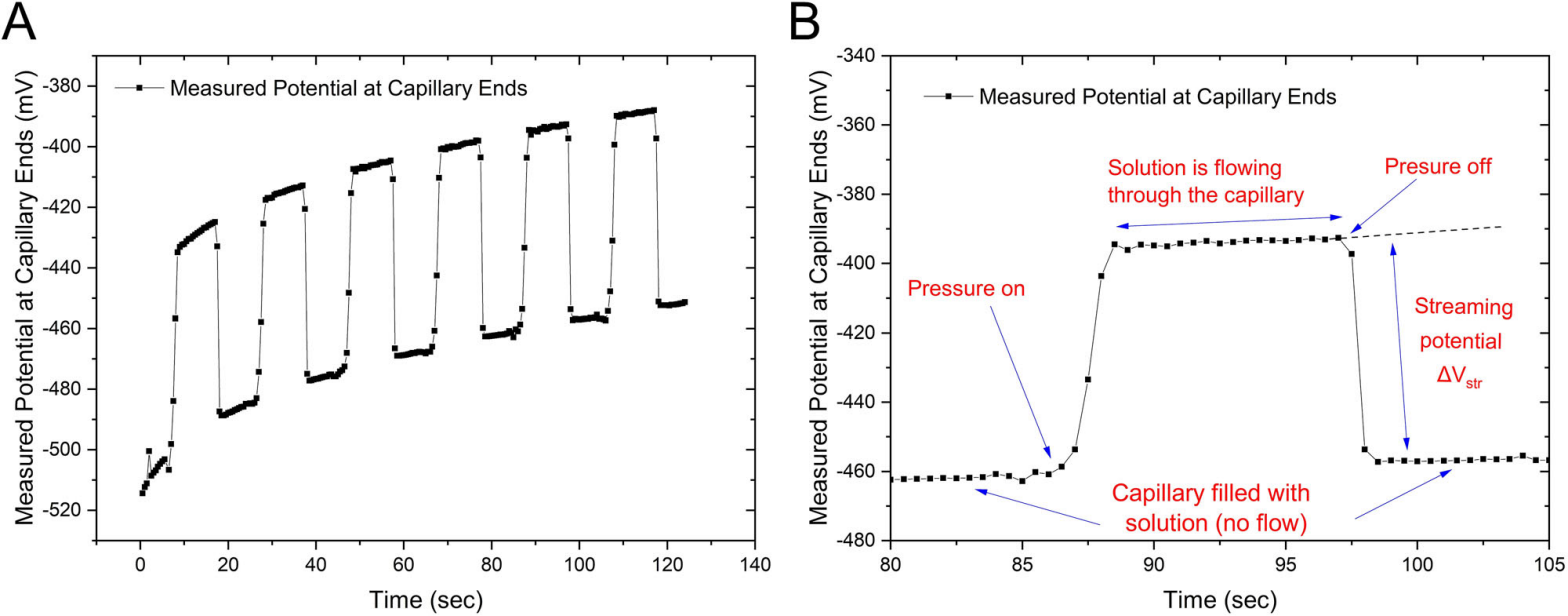
(A) Streaming potential measurement performed by the instrument. (B) Details of an individual streaming potential measurement performed by the instrument. The pressure inside the capillary is rapidly increased from 0 to 2000 mbar and held for a period of about 10 s, followed by a rapid decrease of pressure from 2000 to 0 mbar. The difference in potentials across the capillary can be measured between the two capillary ends when pressure is turned on and off.

The streaming potential measurement data were collected using Elforun Software (Lumex Instruments). The capillary was first rinsed for 3 min and filled with the solution to be used for streaming potential measurements. The streaming potential measurements begin when the pressure inside the capillary was increased from 0 to 2000 mbar and held for 10 s, followed by 10 s of no pressure, and this was then repeated for five more times, with a total cycle lasting about 130 s (Figure 2A). The potential difference was measured between the two capillary ends during this process (Figure 2B). An averaged streaming potential value from five measurements (No. 2–No. 6) was then used for the calculations (*n* = 5). The first measured streaming potential value (0–20 s) in Figure 2A was not used in the averaging due to disturbance at the baseline at the beginning of the analysis.

The potential difference, $\Delta V_{\mathrm{str}},$ can be calculated by the Helmholtz–Smoluchowski equation [23, 24]:

(1) $$\Delta V_{\mathrm{str}}=\frac{\;\Delta P\;\varepsilon \;\varepsilon_{0\;}\zeta \;}{\eta \;\kappa \;4\pi }$$

where Δ*P* is the pressure difference between the inlet and outlet of the capillary, ε is the electrical permittivity of the solution, $\varepsilon_{0}$ is the electrical permittivity of vacuum, ζ is the zeta potential of the capillary inner surface, *η* is the viscosity of the solution, and κ is the solution conductivity. Equation (1) describes the relationship between the potential difference and the fluid pressure between the outlet and the inlet of a capillary with a charged surface (see Ref. [24] for the derivation of this equation).

### 2.5 Viscosity and Conductivity Measurement

Viscosity values of SDS solutions were measured by injecting a plug of thymidine into the capillary and monitoring the time required for the plug to reach the detector window when a 95 mbar pressure was applied to the inlet of the capillary (see Supporting Information section for details). Conductivity values of SDS solutions were obtained by calculating the electrical resistance of the SDS solution in the capillary using the measured current values with the voltage applied (see Supporting Information section for details). The conductivity of deionized water was measured to be 0.02 1/Ω m.

## 3 Results and Discussion

### 3.1 CMC Determination of SDS Using Streaming Potential and Conductivity Measurements

**FIGURE 3:**
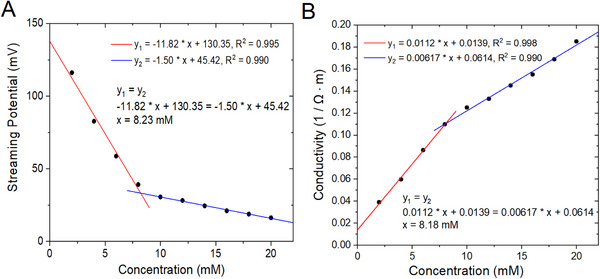
(A) Streaming potentials of SDS solutions measured as a function of concentration. The CMC was calculated to be 8.23 mM. (B) Conductivities of SDS solutions as a function of concentration from the intersection of *y*_1_ (red line) and *y*_2_ (blue line) linear fits. The CMC was calculated to be 8.18 mM.

Figure 3A shows the streaming potential of SDS (anionic surfactant) solutions plotted as a function of SDS concentration. Around the CMC, the properties of the solution showed sharp changes that can be detected by measuring the streaming potentials before and after the CMC. An inflection point was observed around the CMC value in the streaming potential versus concentration plot of the surfactant. The CMC values were determined by the intersections of the two linear regions of the plot: before the CMC, which corresponds to the monomeric state of the surfactant, and after CMC, which corresponds to micellar state of the surfactant [10, 11, 12, 13, 14, 29, 30, 31, 32]. A CMC value of 8.23 mM was calculated by solving for *x* and *y* of the two linear equations. To compare the CMC value of SDS using conductivity measurements, Figure 3B shows conductivity of SDS solutions plotted as a function of its concentration. The CMC value of 8.18 mM was obtained by solving the two linear equations, and the result is similar to the CMC value determined in Figure 3A with streaming potential measurements. The determined CMC value of SDS is in good agreement with CMC values of SDS found in the literature (see Table 1).

**TABLE 1:** Summary of critical micelle concentration (CMC) values measured in this work and comparison to CMC values found in the literature for sodium dodecyl sulphate (SDS), cetyltrimethylammonium bromide (CTAB), CHAPS and Triton X‐100 at 25°C.

Surfactant (type)	CMC values measured in this work at 25°C (mM)	Literature data	
		CMC (mM)	References
SDS (anionic)	8.23 (in water)	7.9–8.2	[10, 11, 12, 13]
CTAB (cationic)	0.93 (in water)	0.8–1	[10, 12, 13]
CHAPS (zwitterionic)	5.80 (in water)	5.4–11	[29, 30, 31, 32]
Triton X‐100 (nonionic)	0.16 (in 15 mM NaCl)	0.19–0.27	[10, 12, 14]

### 3.2 CMC Determination of CTAB, CHAPS and Triton X‐100

**FIGURE 4:**
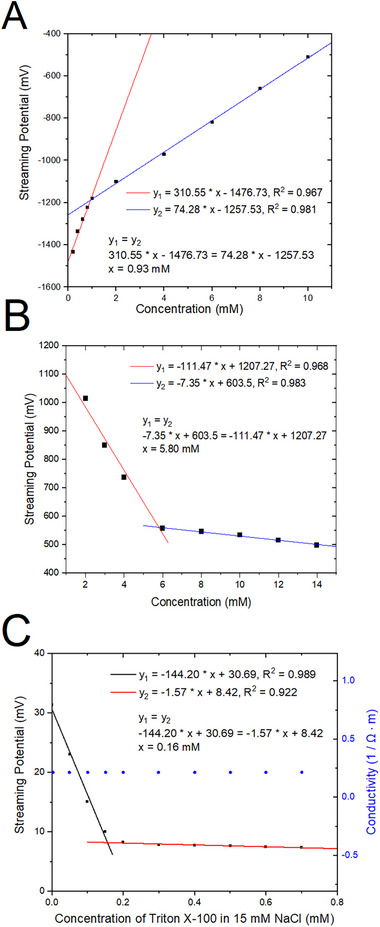
(A) Streaming potentials of CTAB solutions measured as a function of concentration. The CMC was calculated to be 0.93 mM. (B) Streaming potentials of CHAPS solutions measured as a function of concentration. The CMC was calculated to be 5.80 mM. (C) Streaming potentials of Triton X‐100 solutions measured as a function of concentration (black squares). The CMC was calculated to be 0.16 mM. Conductivities of Triton X‐100 solutions were also measured as a function of concentration (blue circles). Triton X‐100 solutions were prepared in 15 mM NaCl.

Figure 4A–C shows the determination of CMC of CTAB (cationic surfactant), CHAPS (zwitterionic surfactant) and Triton X‐100 (non‐ionic surfactants) using the streaming potential measurements. Compared to Figure 3A, where streaming potentials of SDS solutions decreased in magnitude with increased SDS concentration (SDS is an anionic surfactant and has a negative net charge inside the capillary when dissolved in water), streaming potentials of CTAB showed negative streaming potential that increased in magnitude with increased CTAB concentration (CTAB is a cationic surfactant and has a positive charge net inside the capillary when dissolved in water). Similar to Figure 3A, an inflection point was observed in the streaming potential measurements around the CMC value due to the change in the properties of the solution. The CTAB CMC was calculated to be 0.93 mM by solving the two linear equations before and after the CMC. Figure 4B shows the streaming potential as a function of concentration for CHAPS, a zwitterionic surfactant. Similar to SDS (Figure 3A), the streaming potentials of CHAPS solutions decreased in magnitude with increased CHAPS concentration (CHAPS has a net negative charge inside the capillary when dissolved in water). The CMC of CHAPS value was calculated to be 5.80 mM. In Figure 4C, the streaming potential of Triton X‐100 solutions prepared in 15 mM NaCl was plotted as a function of concentration and showed a change of slope at about 0.16 mM. Triton X‐100 molecules are uncharged, and therefore, the surfactant solutions are nonconductive inside the capillary. As a result, there will be no potential difference across a capillary tube when it is forced through from the inlet to the outlet with a pressure (streaming potential value is 0). The role of 15 mM NaCl is to provide ionic strength to the solution. However, with conductivity measurements, no change of slope was observed because the concentration of NaCl is constant. This demonstrates that streaming potential measurements can be used to measure the CMC value of non‐ionic surfactants, when conductivity measurements cannot be used. The determined CMC values of SDS, CTAB and Triton X‐100 are in good agreement with CMC values found in the literature (see Table 1).

### 3.3 Effect of Temperature on CMC Values of SDS and CTAB

**FIGURE 5:**
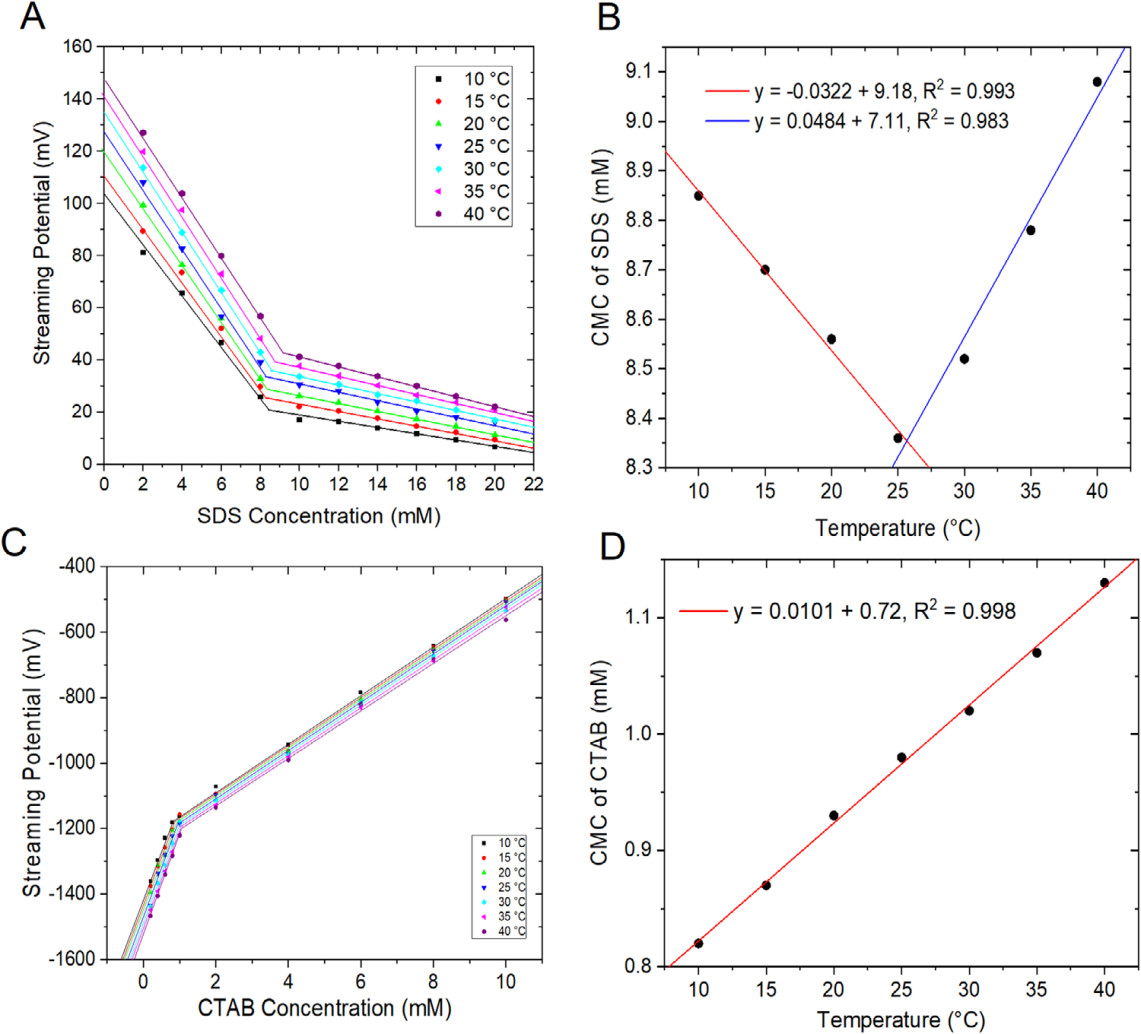
(A) Streaming potentials of SDS solutions measured as a function of concentration at 10°C (black squares), 15°C (red circles), 20°C (green up triangles), 25°C (blue down triangles), 30°C (cyan diamonds), 35°C (pink left triangles) and 40°C (purple hexagons). CMC values were calculated to be 8.85, 8.70, 8.56, 8.36, 8.52, 8.78 and 9.08 mM for 10°C, 15°C, 20°C, 25°C, 30°C, 35°C and 40°C, respectively. (B) CMC values versus temperature for SDS (black circles) obtained from Figure 3A. (C) Streaming potentials of CTAB solutions measured as a function of concentration at 10°C (black squares), 15°C (red circles), 20°C (green up triangles), 25°C (blue down triangles), 30°C (cyan diamonds), 35°C (pink left triangles) and 40°C (purple hexagons). CMC values were calculated to be 0.82, 0.87, 0.93, 0.98, 1.02, 1.07 and 1.13 mM for 10°C, 15°C, 20°C, 25°C, 30°C, 35°C and 40°C, respectively. (D) CMC values versus temperature for CTAB (black circles) obtained from Figure 3C. CMC, critical micelle concentration; CTAB, cetyltrimethylammonium bromide; SDS, sodium dodecyl sulphate.

To illustrate the degree to which CMC is dependant on temperature, Figure 5A,C shows the measured streaming potentials of SDS and CTAB at temperature values of 10°C–40°C, respectively (see Tables S1–S4 for streaming potentials and CMC values). From Figure 5A, the streaming potentials were measured to be about 81–7 mV when the SDS concentration changed from 2 to 20 mM at the lowest studied temperature of 10°C and about 128–22 mV at the highest studied temperature of 40°C for SDS solutions of the same concentration range. Figure 5C shows a plot of CMC value of SDS plotted as a function of temperature. Two linear regions were observed, with decreasing CMC value from 10°C to 25°C, and above 25°C, the CMC was observed to increase. In the first linear region, from 10°C to 25°C, the decrease in CMC with increased temperature is counter intuitive because one would expect that the spontaneous formation of SDS aggregates is exothermic. Increasing the temperature should have made the aggregation process more difficult, thus requiring higher monomer concentration to form micelles. The decrease in CMC with the increase in temperature suggests that at temperatures lower than 25°C, the net change in entropy for the SDS micelle formation process is positive. Positive entropy may suggest that micelle formation is mainly driven by hydrophobic interactions between its amphiphilic monomers, which breaks the three‐dimensional hydrogen‐bonded structure of liquid water [33, 34, 35, 36]. The increase in temperature makes the total Gibbs energy change for micelle formation more negative, requiring lower SDS monomer concentration for micelle formation. Above 25°C, the CMC increases as temperature increases. This may be because the lipophobic property of water decreased at higher temperature, reducing the entropy advantage of micelle formation. The reduction of hydrophobic interactions between the alkyl groups is another factor that can contribute to the increased CMC at higher temperature. The inflection point of the CMC versus T plot at about 25°C is consistent with what has been reported in the literature [33, 34, 35, 36]. Shown in Figure 5C are streaming potentials measured for CTAB solutions with concentrations of 0.2–10 mM at temperature values of 10°C–40°C. From Figure 5C, the streaming potentials were measured to be about −1361 to −499 mV at the lowest studied temperature of 10°C for CTAB concentrations of 0.2–10 mM and −1466 to −562 mV at the highest studied temperature of 40°C for the CTAB solutions. Figure 5D shows the CMC value of CTAB plotted as a function of temperature. The CMC of CTAB increased linearly from 10°C to 40°C, whereas the CMC value of SDS decreased linearly from 10°C to 25°C and then increased linearly from 25°C to 40°C. This is consistent with the observation that the exothermic nature of hydrophobic interaction in aqueous solutions is the driving force for CTAB micelle formation. Alkyl interactions of CTAB are stronger than SDS because CTAB has a longer alkyl chain than SDS [37, 38, 39, 40]. This demonstrates that the CMC value is highly dependant on the properties of the surfactant and may increase or decrease when the temperature is changed.

### 3.4 Zeta Potential and Surface Charge Density Calculations

**FIGURE 6:**
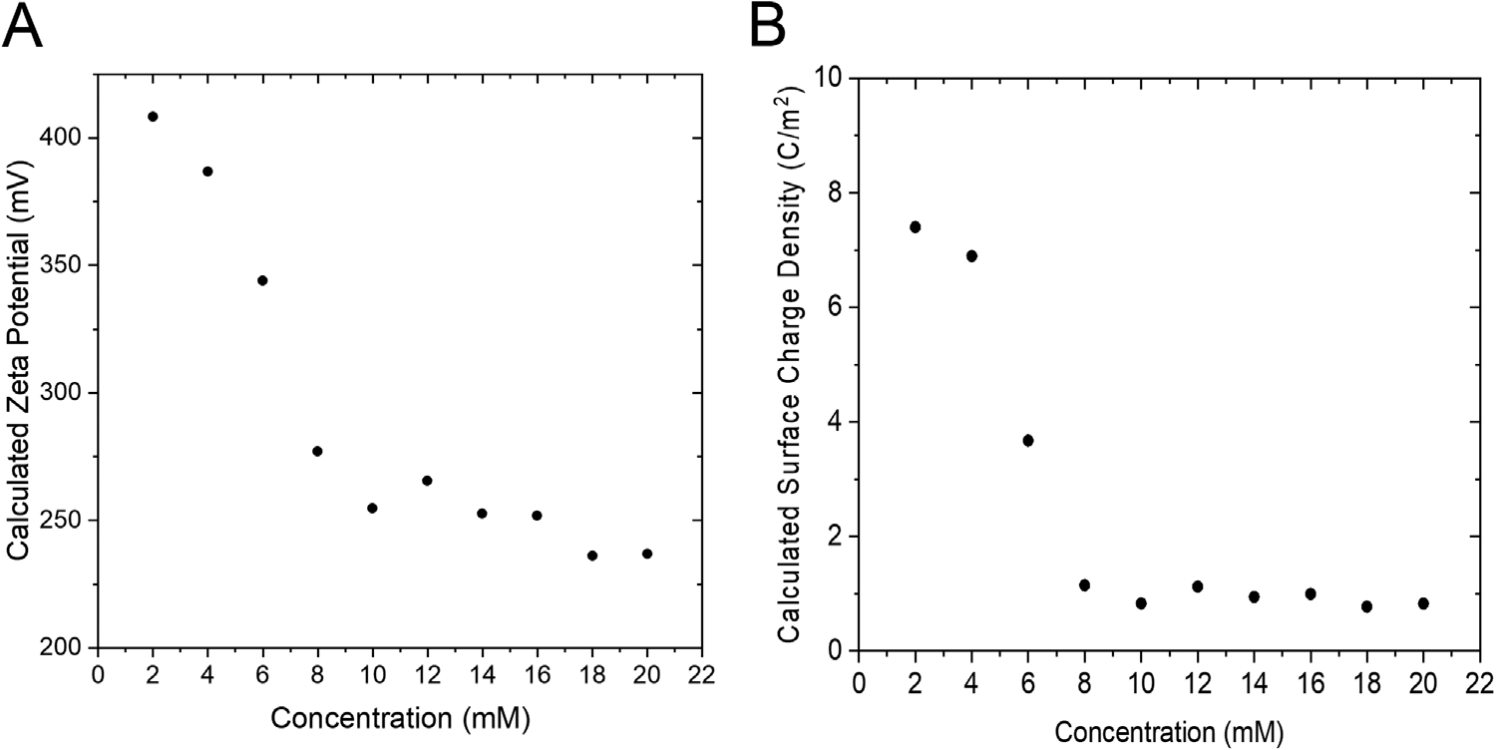
(A) Calculated zeta potentials of SDS determined using measured streaming potentials and conductivities from Figure 3A,B, respectively, and corresponding viscosity values (see Supporting Information section for details of how viscosities were measured). (B) Calculated surface charge density using zeta potentials calculated in Figure 6A.

Zeta potential can be calculated from the measured streaming potentials, viscosities and conductivities according to Equation (1). Figure 6A shows the zeta potential values calculated using the measured streaming potentials in SDS solutions of 2–20 mM as shown in Figure 3A. Streaming potential is highly dependant on the zeta potential of the capillary wall and the conductivity and viscosity of the solution [24]. The corresponding conductivity and viscosity values are reported in Table S5. Both conductivity and viscosity are proportional to the concentration of the electrolyte in the capillary. The zeta potentials were calculated to be about 394–237 mV for SDS solutions with concentrations of 2–20 mM. The zeta potential was observed to reach a plateau at about 8 mM SDS, which is consistent with its CMC (see Figure 3A). Surface charge density was also calculated using the zeta potentials obtained in Figure 6B. The following equation was used to calculate the surface charge density, σ [41]:

(2) $$\sigma ={\left(8\varepsilon \varepsilon_{0}kTC_{\mathrm{bulk}}\right)}^{\frac{1}{2}}\;\sinh\left(\frac{e\psi }{2kT}\right)$$

where ε is the electrical permittivity of the solution, $\varepsilon_{0}$ is the electrical permittivity of vacuum, k is the Boltzmann constant, *T* is the temperature of the solution, $C_{\mathrm{bulk}}$ is the concentration of the bulk solution, e is the elementary electric charge, and ψ is the electrical potential. Equation (2) can also be rewritten as follows:

(3) $$\sigma ={\left(8\varepsilon \varepsilon_{0}RTC_{\mathrm{bulk}}\right)}^{\frac{1}{2}}\;\sinh\left(\frac{zF\psi }{2RT}\right)$$

where z is the number of electrons, F is the Faraday constant, and R is the ideal gas constant. Surface charge densities were calculated to be about 5.6–0.8 C/m^2^ for SDS solutions with concentrations of 2–20 mM. The surface density was also observed to reach a plateau at about 8 mM, which is also consistent with the CMC of SDS. The calculated zeta potentials and surface charge densities reported in this work (Table S5) may be important in future modelling studies for the characterization of silica surfaces.

## 4 Concluding Remarks

By using a CE system capable of measuring streaming potentials, we were able to obtain the CMC values of ionic and non‐ionic surfactants in aqueous solutions. Using the measured streaming potentials, the CMC values of SDS, CTAB, CHAPS and Triton X‐100 were calculated to be 8.23, 0.93, 5.80 and 0.16 mM, respectively, at a temperature of 25°C. In addition, streaming potentials were used to determine the CMC values of SDS and CTAB at temperatures of 10°C–40°C. The effect of temperature on the CMC is different for SDS and CTAB. The CMC value of SDS decreased from 10°C to 25°C and increased from 25°C to 40°C, whereas for CTAB, the CMC only increased linearly from 10°C to 40°C. This shows that the CMC value is highly dependant on the properties of the head groups and alkyl chains of the surfactant, depending on the driving force for micelle formation. Zeta potentials for SDS solutions were calculated from the measured streaming potentials, conductivities and viscosities. The surface charge densities of SDS solutions were calculated from the zeta potentials. The measured streaming potentials and zeta potentials, as well as the surface charge densities calculated in this work, should be important to future modelling studies for the interaction of surfactants with silica surfaces. The results obtained in this work are comparable to results obtained from other CMC determination techniques such as surface tension and conductometric measurements. This work demonstrates that streaming potentials can be used to determine the CMC of cationic, anionic and non‐ionic surfactants, which can be advantageous over other methods because zeta potentials and surface charge densities can also be calculated from measured streaming potentials, conductivities and viscosities.

## Conflicts of Interest

The authors declare no conflicts of interest.

## Supporting information



Supporting Information

## Data Availability

The data that support the findings of this study are available from the corresponding author upon reasonable request.
