# Supplementary material for: Determination of Critical Micelle Concentration of Ionic and Non‐Ionic Surfactants by Streaming Potential Measurements
Source: Electrophoresis. 2025 Apr 23;46(13-14):990–7. doi: 10.1002/elps.8145 (PMC12449752; doi:10.1002/elps.8145)
Supplement: Supplementary file 1 — Supporting Information [file ELPS-46--s001.docx]

**Supporting Information**

**Determination of critical micelle concentration of ionic and nonionic surfactants by streaming potential measurements**

Yuri Chenyakin and David Da Yong Chen*

Department of Chemistry, University of British Columbia, 2036 Main Mall, Vancouver, BC, Canada V6T 1Z1

*Correspondence should be addressed to the following author:

David Da Yong Chen

Department of Chemistry

University of British Columbia

2036 Main Mall, Vancouver, BC, Canada V6T 1Z1

E-mail: chen@chem.ubc.ca

**Viscosity Measurements**

Viscosity measurements of 0 mM, 10 mM, 20 mM, 50 mM and 100 mM SDS bulk solutions inside the capillary, independent of the state of the inner wall, were measured using a Capel-205 capillary electrophoresis system. The temperature inside the capillary was maintained at 25 °C using a capillary thermostat system. A thymidine plug was injected into the inlet of the capillary at 50 mbar for 1 second and a constant pressure of 95 mbar was applied to push the sample plug through the capillary. Viscosity was then calculated using Poiseuille’s equation [1]:

$\eta= \frac{\Delta P r^{2} t}{8 L_{c} L_{d}}$ (1)

where $\Delta P$ is the pressure applied to the capillary, r and $L_{c}$ are the radius and length of the capillary, $L_{d}$is the effective length of the capillary (from inlet to detector) and t is the time required for the sample plug to travel from the inlet to the detector when a pressure $\Delta P$ is applied. To account for capillary diameter variations and uncertainties in the length of the capillary, the accuracy of the pressure and temperature, and the time required for the pressure to reach 95 mbar, a relative viscosity value was calculated based on the known viscosity of pure water [1]:

$\eta= \frac{\eta_{H2O} t}{t_{H2O}}$ (2)

where $\eta_{H2O}$ and $t_{H2O}$ are the viscosity and time values for water, and $\eta$ and $t$ are the viscosity and time values of the solution to be measured. A standard curve was then fitted to the viscosities measured for SDS solutions at 25 °C (Figure S1).

**Conductivity Measurements**

Conductivity measurements of 0 mM, 10 mM, 20 mM, 50 mM and 100 mM SDS bulk solutions inside the capillary, independent of the state of the inner wall, were measured using a Capel-205 capillary electrophoresis system. The capillary was rinsed for 3 minutes at 1000 mbar and filled with the HCl solution to be used for conductivity measurements. With the inlet and outlet capillary ends immersed in the solution (analysis mode), voltages of 1, 5, 10, 15, 20, 25 and 30 kV were applied and the current passing through the capillary was recorded at each voltage. The temperature inside the capillary was maintained at 25 °C using a capillary thermostat system. Electrical resistance can be calculated by using the slope of the applied voltage versus current plot (Figure S2), the area and the length of the capillary. The following equation was used to calculate resistance, $R$ [2]:

$R=Slope of\frac{V_{applied}}{I_{measured}} plot x\frac{\pi r^{2}}{L_{c}}$ (3)

where $r$ and $L_{c}$ are the radius and length of the capillary, respectively. The conductivity of the solution, $\kappa$, is the inverse of the resistance [2]:

$\kappa=\frac{1}{R}$ (4)

The conductivities of 2, 4, 6, 8, 10, 12, 14, 16, 18 and 20 SDS HCl solutions were measured at 25 °C.

Table S1: Streaming potentials of SDS measured at 10, 15, 20, 25, 30, 35 and 40 °C.

| **Streaming potentials at different temperatures values** | | | | | | |  |
| --- | --- | --- | --- | --- | --- | --- | --- |
| **SDS Conc (mM)** | **10 °C** | **15 °C** | **20 °C** | **25 °C** | **30 °C** | **35 °C** | **40 °C** |
| 2.00 | 81.2 | 89.3 | 99.3 | 112.1 | 116.2 | 119.9 | 128.2 |
| 4.00 | 65.6 | 73.6 | 76.5 | 82.7 | 88.9 | 97.5 | 103.9 |
| 6.00 | 46.7 | 52.1 | 55.9 | 57.1 | 66.7 | 73.0 | 79.8 |
| 8.00 | 25.8 | 29.8 | 32.8 | 39.2 | 43.1 | 48.2 | 56.9 |
| 10.00 | 17.1 | 22.1 | 26.2 | 30.6 | 33.7 | 37.7 | 41.2 |
| 12.00 | 16.4 | 20.4 | 23.7 | 28.2 | 30.6 | 33.9 | 37.8 |
| 14.00 | 14.0 | 17.7 | 20.5 | 23.8 | 26.8 | 30.3 | 33.7 |
| 16.00 | 11.7 | 14.6 | 17.5 | 20.5 | 24.3 | 26.5 | 30.1 |
| 18.00 | 9.4 | 12.3 | 14.7 | 18.2 | 20.9 | 23.7 | 26.2 |
| 20.00 | 6.8 | 9.5 | 11.3 | 16.3 | 17.0 | 21.1 | 22.2 |

Table S2: Streaming potentials of CTAB measured at 10, 15, 20, 25, 30, 35 and 40 °C.

| **Streaming potentials at different temperatures values** | | | | | | |  |
| --- | --- | --- | --- | --- | --- | --- | --- |
| **CTAB Conc (mM)** | **10 °C** | **15 °C** | **20 °C** | **25 °C** | **30 °C** | **35 °C** | **40 °C** |
| 0.20 | -1360.8 | -1375.2 | -1395.3 | -1433.8 | -1437.3 | -1448.9 | -1465.8 |
| 0.40 | -1295.7 | -1315.4 | -1310.9 | -1336.3 | -1366.2 | -1390.4 | -1405.1 |
| 0.60 | -1228.1 | -1257.9 | -1280.6 | -1279.0 | -1350.6 | -1330.1 | -1340.3 |
| 0.80 | -1181.5 | -1203.2 | -1205.1 | -1222.8 | -1240.1 | -1270.5 | -1282.9 |
| 10.00 | -1161.8 | -1155.6 | -1182.4 | -1185.1 | -1174.2 | -1218.6 | -1220.3 |
| 12.00 | -1071.7 | -1093.3 | -1112.8 | -1095.9 | -1112.9 | -1129.2 | -1135.5 |
| 14.00 | -945.3 | -962.7 | -964.2 | -971.66 | -975.4 | -980.8 | -990.2 |
| 16.00 | -784.2 | -822.9 | -803.7 | -826.1 | -812.9 | -830.7 | -819.7 |
| 18.00 | -641.6 | -645.1 | -652.3 | -656.8 | -672.5 | -688.5 | -681.6 |
| 20.00 | -499.3 | -499.7 | -506.2 | -507.4 | -534.6 | -525.0 | -562.4 |

Table S3: CMC values of SDS measured in this work at 10, 15, 20, 25, 30, 35 and 40 °C compared to literature data.

| **T (°C)** | **CMC values measured in this work** | **Literature Data** | |
| --- | --- | --- | --- |
|  |  | **CMC (mM)** | **Reference** |
| 10 | 8.85 | 8.8-9.1 | [3–5] |
| 15 | 8.70 | 8.1-8.7 | [3–5] |
| 20 | 8.56 | 8.0-8.5 | [3–5] |
| 25 | 8.36 | 7.7-8.4 | [3–5] |
| 30 | 8.52 | 7.5-8.6 | [3–5] |
| 35 | 8.78 | 7.4-8.6 | [3–5] |
| 40 | 9.08 | 7.9-8.9 | [3–5] |

Table S4: CMC values of CTAB measured in this work at 10, 15, 20, 25, 30, 35 and 40 °C compared to literature data.

| **T (°C)** | **CMC values measured in this work** | **Literature Data** | |
| --- | --- | --- | --- |
|  |  | **CMC (mM)** | **Reference** |
| 10 | 0.82 | N/A | N/A |
| 15 | 0.87 | N/A | N/A |
| 20 | 0.93 | 0.95 | [6] |
| 25 | 0.98 | 0.95-0.97 | [6,7] |
| 30 | 1.02 | 1.08 | [6] |
| 35 | 1.07 | 1.12-1.14 | [6,7] |
| 40 | 1.13 | 1.18 | [6] |

Table S5: Measured streaming potentials, corresponding conductivities and viscosities and calculated zeta potentials of SDS at 25 °C. Dielectric constant of 78.304 was used for zeta potential calculations [8].

| **SDS Concentration (mM)** | **Streaming Potential (mV)** | **Conductivity (1/Ω·m)** | **Viscosity (Pa·s)** | **Calculated Zeta Potential (mV)** | **Calculated Surface Charge Density (C/m^2^)** |
| --- | --- | --- | --- | --- | --- |
| 2.00 | 112.1 | 0.0390 | 9.934E-04 | 394.2 | 5.63 |
| 4.00 | 82.5 | 0.0597 | 9.978E-04 | 386.8 | 6.90 |
| 6.00 | 58.6 | 0.0865 | 1.002E-03 | 344.1 | 3.67 |
| 8.00 | 39.2 | 0.110 | 1.007E-03 | 276.9 | 1.15 |
| 10.00 | 30.8 | 0.125 | 1.011E-03 | 254.6 | 0.83 |
| 12.00 | 28.0 | 0.133 | 1.015E-03 | 265.4 | 1.13 |
| 14.00 | 23.6 | 0.145 | 1.020E-03 | 252.6 | 0.95 |
| 16.00 | 21.1 | 0.155 | 1.024E-03 | 251.9 | 1.00 |
| 18.00 | 17.9 | 0.169 | 1.029E-03 | 235.9 | 0.78 |
| 20.00 | 16.4 | 0.185 | 1.033E-03 | 236.7 | 0.83 |

**Figure S1:** Standard curves of measured viscosities of SDS at 25 °C (red circles). The linear fits shown in the plot were used to calculate the viscosities given in Tables S3.

**Figure S2:** Example of applied voltage versus measured current plot used for the determination of conductivity at a specific concentration (10 mM SDS at 25 °C in this case).

**References**

[1] Peng X, Bowser MT, Britz-McKibbin P, Bebault GM, Morris JR, Chen DDY. Quantitative description of analyte migration behavior based on dynamic complexation in capillary electrophoresis with one or more additives. Electrophoresis 1997. doi:10.1002/elps.1150180509.

[2] Bockris JO, Reddy AKN, Gamboa-Aldeco M. Modern Electrochemistry: Fundamentals of Electrodics. In: *Fundam. Picosci.* 2000 doi:10.1201/b15523.

[3] Goddard ED, Benson GC. CONDUCTIVITY OF AQUEOUS SOLUTIONS OF SOME PARAFFIN CHAIN SALTS. Can J Chem 1957; 35. doi:10.1139/v57-134.

[4] Paula S, Sues W, Tuchtenhagen J, Blume A. Thermodynamics of Micelle Formation as a Function of Temperature: A High Sensitivity Titration Calorimetry Study. J Phys Chem 1995; 99: 11742–11751.

[5] Chatterjee A, Moulik SP, Sanyal SK, Mishra BK, Puri PM. Thermodynamics of Micelle Formation of Ionic Surfactants:  A Critical Assessment for Sodium Dodecyl Sulfate, Cetyl Pyridinium Chloride and Dioctyl Sulfosuccinate (Na Salt) by Microcalorimetric, Conductometric, and Tensiometric Measurements. J Phys Chem B 2001; 105: 12823–12831.

[6] Abbot V, Sharma P. Investigating thermodynamic, acoustic and spectroscopic parameters of rutin trihydrate with cationic surfactant CTAB in hydro-ethanolic solvent systems. J Mol Liq 2021; 328. doi:10.1016/j.molliq.2021.115489.

[7] Shah SK, Bhattarai A. Interfacial and Micellization Behavior of Cetyltrimethylammonium Bromide (CTAB) in Water and Methanol-Water Mixture at 298.15 to 323.15 K. J Chem 2020; 2020. doi:10.1155/2020/4653092.

[8] Malmberg CG, Maryott AA. Dielectric constant of water from 0 to 100 C. J Res Natl Bur Stand (1934) 1956. doi:10.6028/jres.056.001.
